# Among individuals who die of COVID-19, is the percentage who had diabetes actually higher than in those dying of other viral infections?

**DOI:** 10.21203/rs.3.rs-8808866/v1

**Published:** 2026-02-17

**Authors:** Neha V. Reddy, Virginia Pate, Til Stürmer, Rachel Wong, Jeremy Harper, Jane E Reusch, Kenneth J Wilkins, Jena Tronieri, John B Buse, Steven G. Johnson, Carolyn T. Bramante

**Affiliations:** Mayo Medical School; University of North Carolina at Chapel Hill; University of North Carolina at Chapel Hill; Stony Brook University Renaissance School of Medicine; Owl Health Works; University of Colorado Denver Anschutz Medical Campus; National Institute of Diabetes and Digestive and Kidney Disease; University of Pennsylvania Perelman School of Medicine; University of North Carolina School of Medicine; University of Minnesota Twin Cities; University of Minnesota Medical School

## Abstract

**Background:**

Early in the COVID-19 pandemic, mainstream news outlets sensationalized that 30–40% of all coronavirus deaths in the United States occurred among individuals with diabetes. It was unclear why this would be news-worthy because 30–40% is approximately the prevalence of diabetes in older adult, the age group most at risk for mortality from COVID-19. Thus, we sought to quantify the proportion of decedents from COVID-19 who had diabetes. To understand the proportion in context, we also calculated the proportion of decedents from influenza who had diabetes.

**Methods:**

For assessing COVID-19 decedents who had diabetes, we used the National COVID Cohort Collaborative (N3C) data enclave, a nationally-representative, harmonized, and de-identified electronic health record database. For assessing influenza decedents who had diabetes, we used Medicare data. We restricted the N3C sample to > 65 years to align with Medicare eligibility.

**Results:**

Among seniors with inpatient mortality due to COVID-19, 46.6% (95% CI: 46.1–47.0) had diabetes. Among seniors with inpatient mortality from influenza, the crude percent with diabetes was 61.2%. When age-standardized to match the N3C COVID-19 data, the percentage of influenza decedents with diabetes was 63.1% (95% CI: 59.1–67.1).

**Conclusions:**

Among seniors with inpatient mortality from respiratory viruses, a very large proportion had diabetes before infection: 63% of influenza decedents and 47% of COVID-19 decedents. Thus, a high proportion of decedents having diabetes is not new or unique to COVID-19. These findings highlight the value of using available data to contextualize health communication to the public.

## BACKGROUND

In 2022, several mainstream news outlets headlined that 30 to 40% of individuals dying from COVID-19 in the United States had diabetes.^1^ It is unclear why this claim made headlines because 30–40% is the approximate prevalence of diabetes in seniors, the group most susceptible to mortality from COVID-19.^2^

In seeking to better understand this proportion, we reviewed the references cited to support this statistic, and they did not support the statement that proportion of decedents from COVID-19 with diabetes was high. The first was a CDC report explaining that underlying conditions were unknown for 54.9% of COVID-19 decedents.^3^ A meta-analysis of 186 observational studies reporting that diabetes, hypertension, obesity and smoking combined contributed to nearly 30% of COVID-19 deaths, stated that the proportion of COVID-19 deaths attributable to diabetes was 8%.^4^ A cohort study at 10 hospitals seeking to quantify risk of death among COVID-19 patients with prediabetes and diabetes reported that the mortality rate among those with diabetes was 27% while it was 15% among those with prediabetes.^5^ The fourth reference reported similar COVID-19 mortality in those without diabetes, with prediabetes, or with diabetes that was medication-managed (by self-report) compared to those with diabetes who were not taking diabetes medication.^6^

Given these discrepancies between the data cited and presentation of the data in popular media, our objective was to quantify the proportion of decedents from COVID-19 who had diabetes. To understand this proportion in context, we also sought to compare it to the proportion of decedents from influenza who had diabetes. We chose influenza as the comparator respiratory virus because of similar indications for testing and risk factors for severe infection, hospitalization, and death.

## METHODS

This analysis was approved by the University of Minnesota institutional review board (STUDY00011578), which provided a waiver of consent. We used the National COVID Cohort Collaborative (N3C) data enclave, a centralized and harmonized database of de-identified electronic health record data from 78 healthcare systems nationally. For assessing influenza decedents who had diabetes, we used Medicare data. We restricted the N3C sample to > 65 years to align with Medicare eligibility.

In both databases, diabetes was defined as any diagnosis code for diabetes or any diabetes medication prescription in the N3C or in Part D claims in the Medicare database with any available look-back.

In the N3C database, death due to COVID-19 was defined as inpatient mortality for the encounter in which someone was admitted for COVID-19, from March 2020 to February 2022. In the Medicare database, death due to influenza was defined as inpatient mortality for the encounter in which someone was admitted for influenza, from January 2017 to December 2017 because this was the most recent Medicare data available.

As age is an important risk factor for mortality, the age distribution of death due to COVID-19 in the N3C was calculated within 5-year age strata from 65 to 90 + years. These age-specific weights were used to multiply the stratum-specific percentage with diabetes for those with in-patient mortality from influenza in Medicare. The average of these products represents the percent of individuals dying of influenza who had diabetes, if those dying from influenza had the same age distribution as those dying from COVID-19.

## RESULTS

Among seniors with inpatient mortality due to COVID-19, 46.6% (95% CI: 46.1–47.0) had diabetes. Among seniors with inpatient mortality from influenza, the crude percent with diabetes was 61.2%. When age-standardized to match the N3C COVID-19 data, the percentage of influenza decedents with diabetes was 63.1% (95% CI: 59.1–67.1), Figure.

## DISCUSSION

This analysis leveraged N3C and Medicare data and found that among seniors with inpatient mortality from respiratory viruses, a very large proportion had diabetes before infection with either virus − 63% of influenza decedents had pre-infection diabetes and 47% of COVID-19 decedents had pre-infection diabetes. Thus, a high proportion of decedents having diabetes is not new or unique to the COVID-19 virus. These findings highlight the importance of using available data to contextualize health communication to the public.

The large proportion of decedents having diabetes highlights the importance of diabetes prevention, diagnosis, and access to treatment. A meta-analysis of adults with type 2 diabetes reported two medications had high certainty of evidence for lower associations with COVID-19 mortality, with a summary relative risk (SRR) of 0.69 (95% CI 0.60–0.79) for metformin and 0.83 (0.71–0.97) for glucagon-like-peptide-1 receptor agonists.^7^ Another study of adults with diabetes reported that metformin was associated with better survival, OR 0.65 (95% CI 0.45–0.93).^8^

Limitations include that this is not a typical analysis – the intent was not to assess diabetes as an independent risk factor for mortality in patients with COVID compared to patients with influenza. Others have shown that diabetes is an independent risk factor for mortality from COVID-19.^9–11^ In contrast, we report the proportion of decedents from COVID-19 who had diabetes and the proportion of decedents from influenza who had diabetes. This analysis did not account for potential confounding factors including presence of other comorbid conditions in the N3C and Medicare populations.^12^ This analysis should not be considered a causal analysis.

## CONCLUSION

Popular media articles implied that the proportion of COVID-19 decedents who had diabetes was unusually high. However, this analysis demonstrates that among seniors with inpatient mortality after respiratory infection, the proportion of COVID-19 decedents who had diabetes was not unusually high. Diabetes affects approximately 29.2% of adults aged 65 and older in the US and is a serious risk factor for mortality from viral infections.^1^ These findings highlight the value of using available data to contextualize health communication to the public.

## Figures and Tables

**Figure 1:**
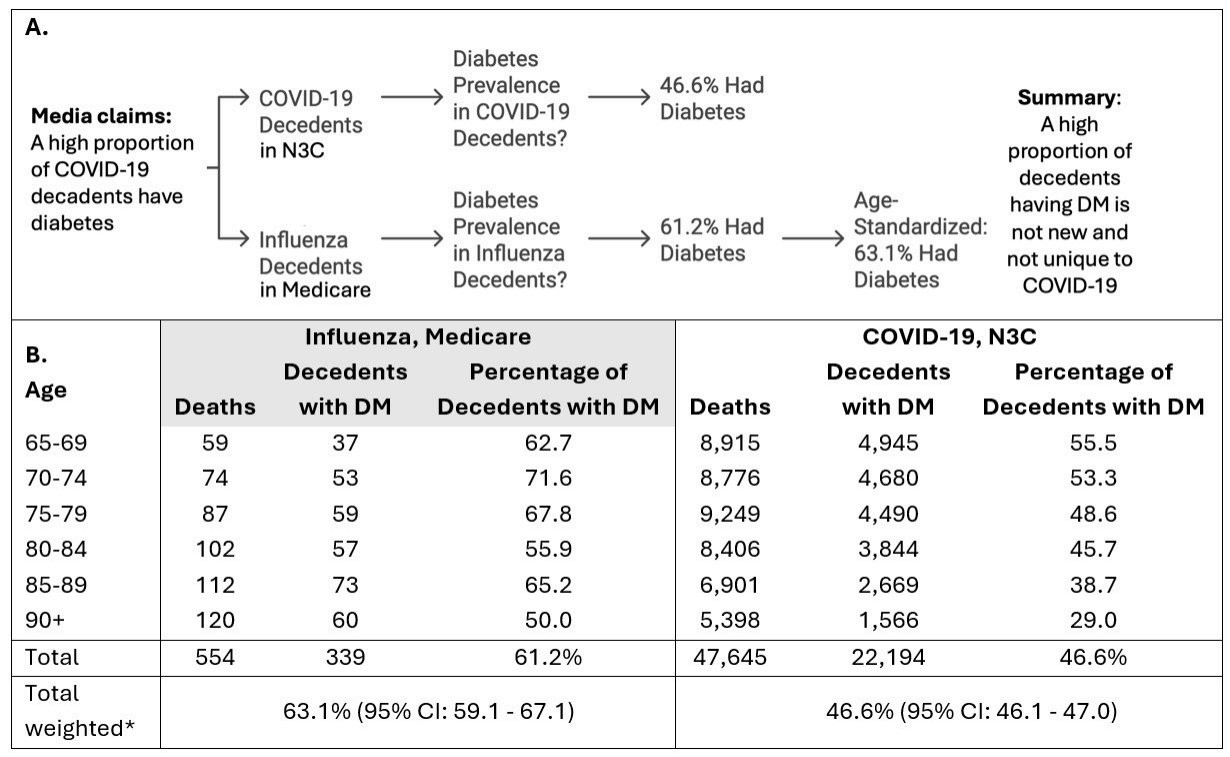
Visual summary of methods used to assess the number of influenza and COVID-19 decedents with diabetes, and the results in each database, Medicare and N3C. **Panel A** is a visual overview of the methods. **Panel B**. gives the number and percentage of decedents with diabetes within each 5-year age category row (65–69; 70–74, etc). The left most column is the 5-year age category and applies to the three Influenza, Medicare columns and the three COVID-19, N3C columns. The overall proportion of decedents is given in the Total row, with the weighted percentage in the bottom row. *DM prevalence in decedents in Medicare weighted to the age distribution of deaths observed in N3C

## Data Availability

The analyses described in this publication were conducted with data or tools accessed through the NCATS N3C Data Enclave https://covid.cd2h.org and N3C Attribution & Publication Policy v 1.2-2020-08-25b supported by NCATS U24 TR002306, Axle Informatics Subcontract: NCATS-P00438-B. This research was possible because of the patients whose information is included within the data and the organizations (https://ncats.nih.gov/n3c/resources/data-contribution/data-transfer-agreement-signatories) and scientists who have contributed to the on-going development of this community resource. The N3C data transfer to NCATS is performed under a Johns Hopkins University Reliance Protocol # IRB00249128 or individual site agreements with NIH. The N3C Data Enclave is managed under the authority of the NIH; information can be found at https://ncats.nih.gov/n3c/resources. The Medicare database infrastructure used for this project was funded by the Pharmacoepidemiology Gillings Innovation Lab (PEGIL) for the Population-Based Evaluation of Drug Benefits and Harms in Older US Adults (GIL200811.0010); the Center for Pharmacoepidemiology, Department of Epidemiology, UNC Gillings School of Global Public Health; the CER Strategic Initiative of UNC’s Clinical and Translational Science Award (UL1TR002489); the Cecil G. Sheps Center for Health Services Research, UNC; and the UNC School of Medicine. The N3C Publication committee confirmed that this manuscript is in accordance with N3C data use and attribution policies; however, this content is solely the responsibility of the authors and does not necessarily represent the official views of the National Institutes of Health or the N3C program.
